# Recent advances in the investigation of curcuminoids

**DOI:** 10.1186/1749-8546-3-11

**Published:** 2008-09-17

**Authors:** Hideji Itokawa, Qian Shi, Toshiyuki Akiyama, Susan L Morris-Natschke, Kuo-Hsiung Lee

**Affiliations:** 1 Natural Products Research Laboratories, School of Pharmacy, University of North Carolina, Chapel Hill, North Carolina 27599-7360, USA

## Abstract

More than 30 *Curcuma *species (Zingiberaceae) are found in Asia, where the rhizomes of these plants are used as both food and medicine, such as in traditional Chinese medicine. The plants are usually aromatic and carminative, and are used to treat indigestion, hepatitis, jaundice, diabetes, atherosclerosis and bacterial infections. Among the *Curcuma *species, *C. longa*, *C. aromatica *and *C. xanthorrhiza *are popular. The main constituents of *Curcuma *species are curcuminoids and bisabolane-type sesquiterpenes. Curcumin is the most important constituent among natural curcuminoids found in these plants. Published research has described the biological effects and chemistry of curcumin. Curcumin derivatives have been evaluated for bioactivity and structure-activity relationships (SAR). In this article, we review the literature between 1976 and mid-2008 on the anti-inflammatory, anti-oxidant, anti-HIV, chemopreventive and anti-prostate cancer effects of curcuminoids. Recent studies on curcuminoids, particularly on curcumin, have discovered not only much on the therapeutic activities, but also on mechanisms of molecular biological action and major genomic effects.

## Background

### Curcuma species

In Asia zingiberaceous plants have been used since ancient times as both spices and medicines, such as in traditional Chinese medicine. Within this plant family, various *Curcuma *species, particularly *C. longa *(turmeric), *C. aromatica *(wild turmeric), and *C. xanthorrhiza *(Javanese turmeric), have been used. The rhizomes of these plants are usually aromatic and carminative, and are used to treat indigestion, hepatitis, jaundice, diabetes, atherosclerosis and bacterial infections [1,2].

**Figure 1:**
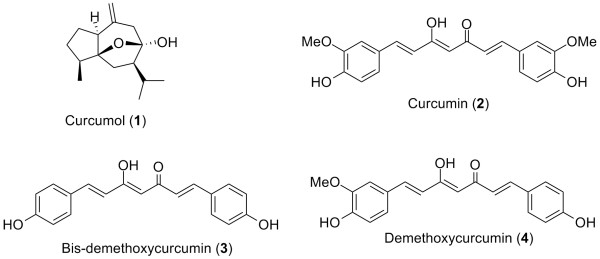
Structures of curcumol and curcuminoids in *Curcuma *species.

Isolated from *Curcuma *plants, various bioactive compounds are useful medicines. For example, curcumol (**1**) (Figure 1), a sesquiterpene isolated from *C. aromatica*, is useful in treating cervical cancer [3].

The rhizomes of *C. longa*, commonly known as turmeric, are used worldwide as spices (e.g. curry), flavoring agents, food preservatives and coloring agents. They are also used as medicines to treat inflammation and sprains in India, China and other Asian countries. Curcuminoids, the main components in *Curcuma *species, share a common unsaturated alkyl-linked biphenyl structural feature and are responsible for their major pharmacological effects. The biological and chemical properties of curcuminoids were reported [4-9].

Curcuminoids in *C. longa *and other *Curcuma *species are mainly curcumin (**2**), bis-demethoxycurcumin (**3**) and demethoxycurcumin (**4**) (Figure 1), among which curcumin is the most studied and shows a broad range of biological activities. This article highlights some of the important biological properties of curcumin and its derivatives, as well as their structure-activity relationships (SAR).

**Figure 2:**
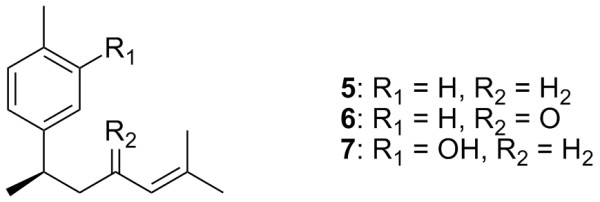
Structure of bisabolane-type compounds in *Curcuma *species.

*C. xanthorrhiza *is used as a tonic in Indonesia and a choleric drug in Europe. Apart from curcuminoids, this species contains bioactive bisabolane-type compounds, such as α-curcumen (**5**), ar-turmerone (**6**) and xanthorrhizol (**7**) (Figure 2). These three compounds demonstrated strong anti-cancer activities against Sarcoma 180 ascites in mice [10-15]. In addition, xanthorrhizol (**7**) exhibited antibacterial activity [16].

### Curcumin and its biological activities

Curcumin (**2**) [diferuloylmethane, 1,7-bis-(4-hydroxy-3-methoxyphenyl)-1,6-heptadiene-3,5-dione] is the main yellow constituent isolated from *C. longa *and other *Curcuma *species. It was first isolated in 1870, but its chemical structure had not been elucidated until 1910 [17] and was subsequently confirmed by synthesis. Curcumin has a unique conjugated structure including two methylated phenols linked by the enol form of a heptadiene-3,5-diketone that gives the compound a bright yellow color.

In addition to its well known anti-inflammatory effects, curcumin also possesses other therapeutic effects on numerous biological targets [18]. Other activities of curcumin include reduction of blood cholesterol level, prevention of low density lipoprotein (LDL) oxidation, inhibition of platelet aggregation, suppression of thrombosis and myocardial infarction, suppression of symptoms associated with type II diabetes, rheumatoid arthritis, multiple sclerosis and Alzheimer's disease, inhibition of human immunodeficiency virus (HIV) replication, enhancement of wound healing, increase of bile secretion, protection from liver injury, cataract formation and pulmonary toxicity and fibrosis, exhibition of anti-leishmaniasis and anti-atherosclerotic properties, as well as prevention and treatment of cancer [18]. Curcumin is non-toxic even at high dosages, and has been classified as 'generally recognized as safe' (GRAS) by the National Cancer Institute [19]. There were also studies focusing on the biology and action mechanisms of curcumin [18,20].

Synthetic bioactive curcumin analogs were developed from the natural compound based on the structure-activity relationship (SAR) studies and optimization of compounds as drug candidates in their relations to different activities, including anti-inflammatory, anti-oxidant, anti-HIV, chemopreventive and anti-cancer (prostate cancer), as well as possible action mechanisms.

### Anti-inflammation

#### Anti-inflammatory activity

Curcumin inhibits the metabolism of arachidonic acid, activities of cyclooxygenase, lipoxygenase, cytokines (interleukins and tumor necrosis factor), nuclear factor-κB (NF-κB) and release of steroids [21]. Curcumin stabilizes lysosomal membranes and causes uncoupling of oxidative phosphorylation. It also possesses strong oxygen radical scavenging activity, which confers anti-inflammatory properties. In various animal studies, a dose of curcumin at 100–200 mg per kilogram of body weight exhibited anti-inflammatory activity. The same dose did not have obvious adverse effects on human systems. Oral median lethal dose (LD_50_) in mice is higher than 2.0 g/kg of body weight [21].

Pro-inflammatory cytokines, such as interleukin-1β (IL-1β) and tumor necrosis factor-α (TNF-α), play key roles in the pathogenesis of osteoarthritis (OA). Anti-inflammatory agents that can suppress the production and catabolic actions of these cytokines may have therapeutic effects on OA and some other osteoarticular disorders. Accordingly, curcumin was examined for its effects on IL-1β and TNF-α signaling pathways in human articular chondrocytes *in vitro *[22]. Expression of collagen type II, integrin β1, cyclo-oxygenase-2 (COX-2) and matrix metalloproteinase-9 (MMP-9) genes was monitored by Western blotting. The effects of curcumin on the expression, phosphorylation, and nuclear translocation of protein components of the NF-κB system were studied with Western blotting and immunofluorescence respectively. The results indicated that curcumin suppressed IL-1β-induced NF-κB activation via inhibition of inhibitory protein κBα (IκBα) phosphorylation, IκBα degradation, p65 phosphorylation and p65 nuclear translocation. Curcumin also inhibited IL-1β-induced stimulation of up-stream protein kinase B Akt. These events correlated with the down-regulation of NF-κB targets, including COX-2 and MMP-9. Similar data were obtained when chondrocytes were stimulated with TNF-α. Curcumin also reversed the IL-1β-induced down-regulation of collagen type II and β1-integrin receptor expression. These results indicate that curcumin may be a naturally occurring anti-inflammatory nutritional agent for treating OA *via *suppression of NF-κB mediated IL-β/TNF-α catabolic signaling pathways in chondrocytes [22]. Curcumin was found to act by diverse anti-inflammatory mechanisms at several sites along the inflammation pathway [23].

#### Anti-inflammatory SAR

**Figure 3:**
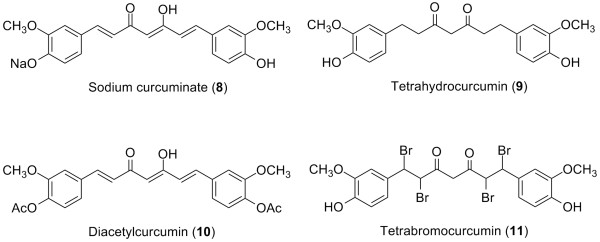
Structures of semi-synthetic analogs tested for anti-inflammatory activity.

The active constituents of *C. longa *are curcuminoids, including curcumin (**2**), demethoxycurcumin (**3**) and bisdemethoxycurcumin (**4**) [24] (Figure 1), among which curcumin is the most potent anti-inflammatory agent [25]. In addition to these natural curcuminoids, sodium curcuminate (**8**) and tetrahydrocurcumin (**9**) (Figure 3) showed potent anti-inflammatory activity at low doses in carrageenin-induced rat paw edema and cotton pellet granuloma assays [26]. Other semi-synthetic analogs of curcumin were screened for anti-inflammatory activity in the same assays; diacetylcurcumin (**10**) and tetrabromocurcumin (**11**) (Figure 3) were the most potent [27,28]. The presence of the β-diketone moiety as a linker between the two phenyl groups was deemed important for the anti-inflammatory activity.

Nurfina *et al*. designed and synthesized 13 symmetrical curcumin analogs (**12**–**24**) [29]. Anti-inflammatory activity was evaluated by inhibition of carrageenin-induced swelling of rat paw (Table 1); and the following SAR conclusions were drawn: (a) appropriate substituents on the phenyl rings were found necessary for anti-inflammatory activity. Unsubstituted compound **12**, *ortho*-methoxy, substituted analog **18**, and *meta*-methoxy substituted analog **13 **showed no inhibitory activity; (b) proper substituents at the *para-*positions of the phenyl rings were also crucial. A *para*-phenolic group leads to the most potent anti-inflammatory activity [compare **3 **(*p*-OH), **21 **(*p*-CH_3_), **20 **(*p*-OCH_3_), **19 **(*p*-Cl) as well as **2 **with **22 **and **24 **with **14**]; and (c) size of the substituents adjacent to a *para*-phenol was found to be important for potency. Dimethyl substitution (**15**) at R_2 _and R_4 _enhanced the activity most, followed by diethyl (**16**) and dimethoxy (**24**). Compound **21 **with two isopropyl moieties showed weaker activity, while **23 **with bulky tetrabutyl substitution at both positions showed no anti-inflammatory activity.

**Table 1:** Anti-inflammatory activity data of curcumin derivatives

**Compound**	**R_1_**	**R_2_**	**R_3_**	**R_4_**	**ED_50 _(mg/kg)**
**2**	H	OCH_3_	OH	H	38 ± 4
**3**	H	H	OH	H	73 ± 5
**12**	H	H	H	H	NA
**13**	H	OCH_3_	H	H	NA
**14**	H	OCH_3_	OCH_3_	OCH_3_	NA
**15**	H	CH_3_	OH	CH_3_	13 ± 2
**16**	H	C_2_H_5_	OH	C_2_H_5_	22 ± 6
**17**	H	i-C_3_H_7_	OH	i-C_3_H_7_	58 ± 21
**18**	OCH_3_	H	H	H	NA
**19**	H	H	Cl	H	NA
**20**	H	H	OCH_3_	H	82 ± 7
**21**	H	H	CH_3_	H	80 ± 18
**22**	H	OCH_3_	OCH_3_	H	50 ± 22
**23**	H	*t*-C_4_H_9_	OH	*t*-C_4_H_9_	NA
**24**	H	OCH_3_	OH	OCH_3_	28 ± 5

NA: not active

ED_50 _values are expressed as 'means ± standard deviations'.

**Figure 4:**
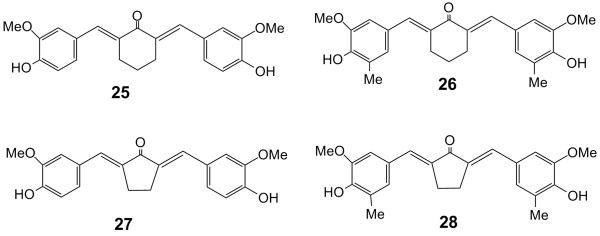
Structures of cyclovalone (**25**) and three related analogs.

Cyclovalone (**25**) and three analogs (**26**–**28**) (Figure 4) having a cyclohexanone or cyclopentanone in the linker between the two phenyl rings showed anti-inflammatory activity to inhibit cyclooxygenase [30]. Compounds **26**–**28 **were more potent than curcumin (**2**) which was used as a reference standard. The dimethylated **28 **and **26 **were more potent than **27 **and **25 **respectively, and thus, the addition of methyl groups on the phenyl rings enhanced anti-inflammatory activity. The increased size of the cycloalkanone ring, by replacing the cyclopentanone in **27 **with a cyclohexanone in **25**, increased inhibitory potency. However, this effect was not seen in the dimethylated compounds **28 **and **26 **respectively, both of which were comparably potent.

**Figure 5:**
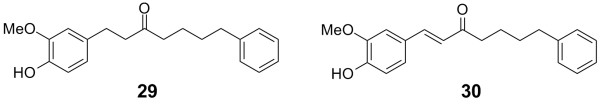
Structures of yakuchinones A (**29**) and B (**30**).

Besides curcumin, other structurally related constituents of plants in the Zingiberaceae family possess anti-inflammatory activity [31]. Examples are the phenolic yakuchinones A and B (**29 **and **30**) isolated from *Alpinia oxyphylla *[32-34] (Figure 5).

### Anti-oxidation

#### Anti-oxidant activity

Most natural anti-oxidants can be classified into two types of compounds, namely phenolic and β-diketone [35]. Sesaminol isolated from sesame belongs to the former, while *n*-triacontane-16,18-dione isolated from the leaf wax of *Eucalyptus *belongs to the latter. Curcumin (**2**) is one of the few anti-oxidants that possess both phenolic hydroxy and β-diketone groups in one molecule. Its unique conjugated structure includes two phenols and an enol form of a β-diketone. Therefore, it may have a typical radical trapping ability and a chain-breaking anti-oxidant activity.

Curcumin is a potent anti-oxidant whose action mechanism is not well understood. However, the nonenzymatic anti-oxidant process of a phenolic compound is generally thought to have two stages as follows:

S-OO• + AH ↔ SOOH + A•

A• + X• → nonradical materials

Where S is the oxidized substance; AH is the phenolic anti-oxidant; A• is the anti-oxidant radical; and X• is another radical species or the same species as A• [35]. While the first stage is reversible, the second stage is irreversible and must produce stable radical terminated compounds. Structural elucidation of the terminated compounds may contribute significantly to understanding the mechanism of the phenolic anti-oxidant. It has recently been shown that dimerization is a main termination process of the radical reaction of curcumin itself. In food, the anti-oxidant coexists with large amounts of oxidizable biomolecules, such as polyunsaturated lipids. These biomolecules were found to produce reactive peroxy radicals during their oxidation, which may act as X• and couple with the anti-oxidant radical (A•) in the second step of the above anti-oxidation scheme [36].

#### Anti-oxidant SAR

Curcumin showed both anti-oxidant and pro-oxidant effects in oxygen radical reactions. Depending on the experimental conditions, it may act as a scavenger of hydroxy radicals or a catalyst in the formation of hydroxy radicals [37-39]. The anti-oxidant effect of curcumin presumably arises from scavenging of biological free radicals.

**Figure 6:**
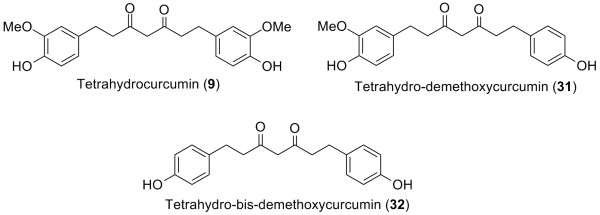
Structures of tetrahydrocurcuminoids.

The anti-oxidant activities of three natural curcuminoids (**2**–**4**) and their hydrogenated analogs (**9**, **31**, **32**) (Figure 6) were examined in three bioassay models, i.e. the linoleic acid auto-oxidation model, rabbit erythrocyte membrane ghost system, and rat liver microsome system. The results obtained from the three models were consistent. Curcumin (**2**) and tetrahydrocurcumin (**9**) had the strongest anti-oxidant activity among the natural and hydrogenated curcuminoids respectively [35]. Among all six compounds, tetrahydrocurcumin (**9**) showed the highest potency, implying that hydrogenation of curcuminoids increased their anti-oxidant ability. Absence of one or both methoxy groups resulted in decreased anti-oxidant activity in both natural curcuminoids and tetrahydrocurcuminoids. In contrast, Sharma *et al*. reported that the presence of methoxy groups in the phenyl rings of curcumin enhanced anti-oxidant activity [40].

Venkatessan *et al*. [41] used three models to investigate the importance of the phenolic hydroxy groups, as well as other substituents on the phenyl rings of curcuminoids, to anti-oxidant activity. The three anti-oxidant bioassays were inhibition of lipid peroxidation, free radical scavenging activity by the DPPH method, and free radical scavenging activity by the ABTS method. The data and compound structures are shown in Table 2. Generally, curcumin analogs with a phenolic moiety were more potent than non-phenolic analogs, and thus, phenolic substitution is important for anti-oxidant activity. Compound **15**, a 4'-hydroxy-3',5'-dimethyl substituted analog, showed potency in all three bioassays. However, compound **23**, a 4'-hydroxy-3',5'-di-*t*-butyl analog, was ten-fold less potent in the lipid peroxidation assay, indicating that steric hindrance at the positions flanking the hydroxyl group decreased anti-oxidative activity. Changing the 3'-methoxy group in curcumin (**2**) to an ethoxy group in **33 **had little effect on anti-oxidant activity, but both compounds were more potent than **3**, which does not have an alkoxy group at the 3'-position. In all three systems, tetrahydrocurcumin (**9**) and curcumin (**2**) showed comparable activity. This result suggests that enhanced electron delocalization of the double bonds may not be essential to anti-oxidant activity of curcuminoids.

**Table 2:** Anti-oxidant activity data of curcumin derivatives

**Compound**	**R_1_**	**R_2_**	**R_3_**	**Lipid peroxidation inhibition IC_50 _(μM)**	**DPPH scavenging IC_50 _(μM)**	**ABTS scavenging TEAC**		
						**3 min**	**9 min**	**15 min**
**2**	OCH_3_	OH	H	1.30	20.02	2.61	3.09	3.37
**3**	H	OH	H	2.19	32.08	3.04	4.31	4.96
**9**	structure formula **9 **above			1.83	18.22	2.08	2.37	2.52
**10**	OCH_3_	OAc	H	1.85	NA	1.33	2.01	2.33
**12**	H	H	H	NA	>250	1.57	2.78	3.36
**14**	OCH_3_	OCH_3_	OCH_3_	15.32	NA	1.90	2.98	3.43
**15**	CH_3_	OH	CH_3_	0.63	21.75	0.89	1.13	1.28
**20**	H	OCH_3_	H	NA	>250	2.05	2.04	2.14
**21**	H	CH_3_	H	NA	>250	0.67	1.52	1.96
**22**	OCH_3_	OCH_3_	H	NA	>250	1.86	2.49	2.67
**23**	*t*-C_4_H_9_	OH	*t*-C_4_H_9_	6.48	23.72	0.81	0.96	1.07
**33**	OC_2_H_5_	OH	H	1.11	30.32	2.36	3.07	3.32
**34**	H	SCH_3_	H	NA	NA below 90	1.09	ND	ND

IC_50 _is the concentration required for 50% inhibition of lipid peroxidation or scavenging of DPPH radical. TEAC is the trolox equivalent anti-oxidation capacity, which is defined as the mM concentration of a trolox solution having the antioxidant capacity equivalent to a 1.0 mM solution of the substance under investigation.

NA: not active

ND: not determined.

The anti-oxidant mechanisms of curcumin have been investigated. The salient finding is that curcumin is a phenolic chain-breaking anti-oxidant, which donates H atoms from the phenolic groups [42-47]. However, some contrasting results suggest that H atom donation takes place at the active methylene group in the diketone moiety [48,49]. Ligeret *et al*. evaluated the effects of curcumin and numerous derivatives on the mitochondrial permeability transition pore (PTP), which can release apoptogenic factors from mitochondria to induce apoptosis [50]. The authors postulated that PTP opening is closely related to the anti-oxidant property of curcumin. Based on the data on mitochondria swelling, O_2_• and HO• production, thiol oxidation and DPPH• reduction, the authors concluded that phenolic groups in curcuminoids are essential for activity, and are more effective at the *para *position than at the *ortho *position. In addition, an electron donating group at the *ortho *position relative to the phenolic group is also required for activity, while *t*-butyl and bulky substituents are not favorable. In contrast, electron-withdrawing substitution, such as NO_2_, reduced activity. Although ferulic acid does not show anti-oxidant effects, replacing the β-diketone moiety of curcumin with a cyclohexanone ring attenuated anti-oxidant activity. Thus, the authors concluded that the β-diketone contributed to, but could not induce, the activity of curcumin derivatives. The conclusions agree with the prevailing SAR for anti-oxidant activity.

**Figure 7:**
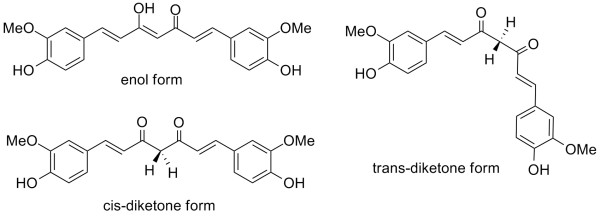
Structures of curcumin conformers.

However, in one study, a curcumin analog without phenolic and methoxy groups was found to be as potent as curcumin in terms of scavenging hydroxy radicals and other redox properties [51]. Wright employed theoretical chemistry to interpret the controversy [52]; taking into account the diversity of test free radicals, solvents, and pH ranges used in the literature. First, he explored the stabilities of curcumin conformers, pointing out that the enol form is the most stable, followed by the *trans*-diketo form, and then the *cis*-diketo form (Figure 7). Calculations showed that the phenolic O-H is the weakest bond in curcuminoids. This theoretical approach favors the necessity of a phenolic OH group for the anti-oxidant activity of curcumin and its analogs. However, the C-H bond of the methylene group becomes active when radicals with high bond dissociation enthalpy, such as methyl and *t*-butoxy radicals, are used. Thus, differences among experimental results can be possibly due to the differences in the attacking radicals used in different bioassay systems.

### Anti-HIV

#### Anti-HIV activity

Oxidative stress is implicated in HIV-infection. It was suggested that plant anti-oxidants may offer protection from viral replication and cell death associated with oxidative stress in patients with HIV/acquired immune deficiency syndrome (AIDS) [53]. Curcumin (**2**) can inhibit purified HIV type 1 integrase, HIV-1 and HIV-2 protease, and HIV-1 long terminal repeat-directed gene expression of acutely or chronically infected HIV-1 cells. Curcumin can also inhibit lipopolysaccharide-induced activation of NF-κB, a factor involved in the activation and replication of HIV-1. However, curcumin did not show significant efficacy in clinical trials.

In addition to the lipid soluble component curcumin, turmeric also contains the water-soluble extract turmerin (molecular weight: 24000 Daltons). Neither turmeric nor turmerin has been studied for anti-HIV activity. In a limited number of studies, cell viability and p24 antigen release by CEMss-T cells infected with HIV-III_B _strain (acute infection model) and proliferative responses of human mononuclear cells derived from HIV patients (chronic infection model) stimulated with phytohematoglutinin, concanavalin A, and pokeweed mitogen were examined in the presence of AZT, curcumin, and turmerin. In infective assays, neither turmerin nor curcumin individually reduced p24 antigen release or improved cell viability [53]. However, AZT (5 μM) plus turmerin (800 ng/ml) inhibited infection by 37% and increased cell numbers by 30%. In the proliferation assay, lymphocytes from HIV-infected patients showed better inhibition of mitogen responsiveness to turmerin (800 ng/ml) than that of AZT at 5 μM or turmerin at 80 ng/ml. Turmerin inhibited HIV-infected T-cell proliferation and, in combination with AZT, decreased T-cell infection and increased cell viability. These data suggest that effective anti-HIV therapy may be possible using lower, less toxic doses of AZT in the presence of turmerin [53].

#### Anti-HIV SAR

In addition to reverse transcriptase and protease, HIV-1 integrase is being explored as a new target for the discovery of effective AIDS treatments. HIV-1 integrase is the enzyme that catalyzes the integration of the double-strained DNA of HIV into the host chromosome [54]. Curcumin inhibited this activity of HIV-1 integrase [54]. Other classes of compounds inhibited HIV-1 integrase in enzyme assays, but few showed specificity against HIV-1 integrase and even fewer were active in cell-based assays [55]. Curcumin was reported to have moderate activity in cell-based assays, in addition to its activity in enzyme assays [56].

Therefore, modified curcumin analogs were developed for anti-HIV potency as well as action mechanism studies [54,57]. Mazumder *et al*. [57] synthesized curcumin analogs (Table 3) as probes to study the mechanism of anti-HIV-1 integrase. Evidence suggests that curcumin does not bind to HIV-1 integrase at either the DNA-binding domain [58] or the binding site of another HIV-1 integrase inhibitor, i.e. NSC 158393 [59]. Compounds without a hydroxy group on the phenyl ring (**12**, **20**) did not inhibit HIV-1 integrase. Therefore, hydroxy groups on the phenyl rings are apparently essential for inhibitory activity. Compounds **35 **and **36**, which contain two and one catechol ring respectively, exhibited much greater activity than curcumin (**2**), indicating that replacing one or both methoxy groups on curcumin with hydroxy groups increased anti-HIV activity. Tetrahydrocurcumin (**9**), with a saturated linker between the phenyl groups, did not show inhibitory activity in this assay, suggesting that an unsaturated linking group also contributed to activity. In addition, compound **37**, with a unique linker bridging two catechol rings, showed potency comparable to that of **35 **and **36**, and greater than that of **2**.

**Table 3:** Anti-HIV integrase activity data of curcumin derivatives

**Compound**	**R_1_**	**R_2_**	**R_3_**	**R_4_**	**3-processing IC_50 _(μM)**	**Strand transfer IC_50 _(μM)**
**2**	OCH_3_	OH	OCH_3_	OH	150	140
**3**	H	OH	H	OH	120	80 ± 20
**4**	H	OH	OCH_3_	OH	140	120
**9**	structure formula **9 **above				>300	>300
**12**	H	H	H	H	>300	>300
**20**	H	OCH_3_	H	OCH_3_	>300	>300
**35**	OH	OH	OH	OH	6.0 ± 1.5	3.1 ± 0.12
**36**	OCH_3_	OH	OH	OH	18.0 ± 9.0	9.0 ± 3.0
**37**	structure formula **37 **above				9 ± 7	4.0 ± 1.5

IC_50 _values are expressed as 'means ± standard deviations'.

**Figure 8:**
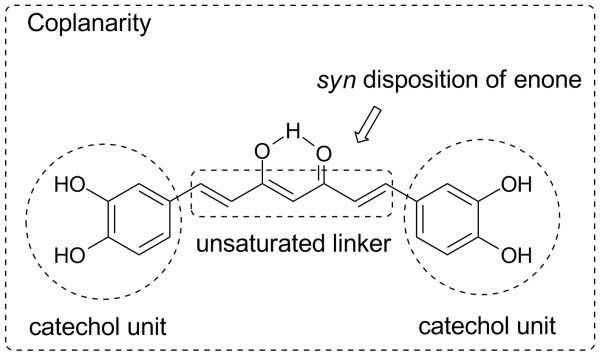
Schematic diagram of structural features favoring anti-HIV-1 integrase activity.

In the further SAR investigation of curcumin analogs as inhibitors of HIV-1 integrase, a *syn *disposition of the C=C=C=O moiety in the linker and a coplanar structure were found to be important to the integrase inhibitory activity of curcumin analogs [55]. The experimental results are consistent with the quantitative structure-activity relationships (QSAR) computed with MOE (Chemical Computing Group, Canada) and Cerius2 (Molecular Simulations, USA) programs [60]. Figure 8 summarizes the anti-HIV-1 integrase SAR of curcumin analogs. However, no therapeutic indices were reported for the tested compounds.

### Chemoprevention

Chemoprevention is a relatively new concept. It attempts to intervene at early stages of cancer before the invasive stage begins [61]. Nontoxic agents are administered to otherwise healthy individuals who may be at increased risk for cancer. Some potential diet-derived chemopreventive agents include epigallocatechin gallate in green tea, curcumin in curry and genistein in soya. Curcumin demonstrated a wide-range of chemopreventive activities in preclinical carcinogenic models of colon, duodenum, fore-stomach, mammary, oral and sebaceous/skin cancers. The National Cancer Institute is conducting Phase I clinical trials of curcumin as a chemopreventive agent for colon cancer [62]. Curcumin's chemopreventive mechanisms are pleiotropic. It enhanced the activities of Phase 2 detoxification enzymes of xenobotic metabolism, including glutathione transferase [63] and NADPH:quinone reductase [64]. It also inhibited pro-carcinogen activating Phase 1 enzymes such as cytochrome P450 1A1 [65]. As regards its mode of chemopreventive action in colon cancer, curcumin exhibited diverse metabolic, cellular and molecular activities including inhibition of arachidonic acid formation and its further metabolism to eicosanoids [66].

### Anti-prostate cancer

Prostate cancer is the most common cancer among males in the West [67] and is a complex heterogeneous disease that affects different men differently. The cause of prostate cancer is largely unknown. However, androgen and the androgen receptor (AR) are postulated to play crucial roles in the development of prostate cancer [68].

Prostate cancer is currently treated with a combination of surgery, radiation and chemotherapy. The therapeutic agents used clinically include steroidal anti-androgens, such as cyproterone acetate, and non-steroidal anti-androgens, such as flutamide and bicartamide. The steroidal anti-androgens possess partial agonistic activity and overlapping effects with other hormonal systems, leading to complications such as severe cardiovascular problems, gynecomastia, libido loss and erectile dysfunction [69-71]. Non-steroidal anti-androgens have fewer side effects and higher oral bioavailability than steroidal anti-androgens.

While non-steroidal anti-androgens are advantageous, anti-androgen withdrawal syndrome was found in patients receiving non-steroidal anti-androgens for several months [72,73]. Long-term drug usage would lead to mutation of the AR, and the non-steroidal anti-androgens may exhibit agonistic activity to the mutant AR [74]. In addition, the clinically available anti-androgens are unable to kill prostate cancer cells, and within one to three years of drug administration, the cancer usually develops into an androgen refractory stage [72-74]. Therefore, new classes of anti-prostate cancer drugs are urgently needed.

Prostate cancer occurs much less frequently in Asia than in the West [75], possibly due to dietary differences. Turmeric is much more highly consumed as both spice and medicine in India, Thailand, China and Japan than in the West. Thus, we and other researchers investigated turmeric and its constituent curcumin for anti-prostate cancer effects.

**Figure 9:**
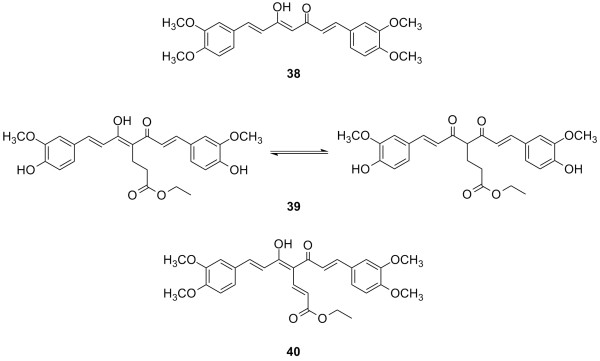
Structures of JC-9 (**38**), ECECur (**39**) and LL-80 (**40) **with anti-prostate cancer activity.

Although curcumin is a well known anti-inflammatory and anti-oxidant agent, its anti-prostate cancer activity has not been extensively explored. Over the last decade, our research group has used curcumin (**2**) as a lead compound for the design and synthesis of curcumin analogs as a new class of potential anti-androgenic agents for the treatment of prostate cancer as well as for action mechanism studies [76-81]. Certain curcumin analogs including **38 **(JC-9), **39 (**4-ethoxycarbonyl curcumin, ECECu) and **40 **(LL-80) (Figure 9), showed potent *in vitro *cytotoxic activity against LNCaP and PC-3 human prostate cancer cell lines (Table 4). Among them, compound **40 **showed the most potent activity, suggesting that introducing a conjugated side chain in the enol-ketone linker may stabilize the enol-ketone form as the predominant tautomer (Figure 9), which may contribute to the anti-prostate cancer activity. Although the entire structure of the AR has not been fully determined and the mechanism of how curcumin derivatives interact with the AR is still unclear, preliminary studies showed that these curcumin derivatives inhibit AR function *via *an AR degradation pathway, which plays an important role in the growth of prostate cancer [82,83]. In addition, compound **38 **(JC-9) with its potent anti-androgenic activity and stable physiological properties was identified as a lead anti-AR compound. Clinical trials against prostate cancer are being planned.

**Table 4:** Cytotoxic activity data of curcumin derivatives against PC-3 and LNCaP prostate cancer cell lines

**Compound**	**R_1_**	**R_2_**	PC-3 IC_50 _(μM)*	LNCaP IC_50 _(μM)*
**2**	H	H	7.7	3.8
**38**	CH_3_	H	1.1	1.3
**39**	H	CH_2_CH_2_COOEt	5.1	1.5
**40**	CH_3_	CH=CHCOOEt	1.0	0.2

IC_50 _values are mean concentrations that inhibit cell growth by 50% (variation between replicates was less than 5%).

IC_50 _values are expressed as 'means'.

We prepared four series of new curcumin analogs [81] including monophenyl curcumin analogs, heterocycle-containing curcumin analogs, curcumin analogs bearing various substituents on the phenyl rings, and curcumin analogs with various linkers, which are being tested for their anti-prostate cancer activity and action mechanism. New curcumin analogs from other research groups [84-86] are also being evaluated for cytotoxic activity against two human prostate cancer cell lines, i.e. LNCaP and PC-3, and inhibitory activity to the AR, with goals to elucidate more refined SAR and optimize curcumin analogs to develop better anti-prostate cancer drugs.

## Conclusion

Natural curcuminoids are compounds found in *Curcuma *species, which are used as a medicine of the upper class of traditional Chinese medicine herbs that are generally not toxic and are in rich content in natural foods and spices. Curcuminoids and other natural and synthetic curcuminoids possess various bioactivities including anti-inflammatory, anti-oxidant, anti-HIV, chemopreventive and anti-prostate cancer effects. In addition, curcumin was recently found to prevent experimental rheumatoid arthritis [87]. Recent studies on curcuminoids, particularly on curcumin, have discovered not only much on the therapeutic activities, but also on mechanisms of molecular biological action and major genomic effects. Our research group developed some anti-androgenic curcumin analogs as anti-prostate cancer agents.

## Abbreviations

AIDS: acquired immune deficiency syndrome; ABTS: 2,2'-azino-bis(3-ethylbenzthiazoline-6-sulphonic acid); AR: androgen receptor; COX-2: cyclo-oxygenase-2; DPPH: 2,2-diphenyl-1-picrylhydrazyl; ECECu: 4-ethoxycarbonyl curcumin; HIV: human immunodeficiency virus; IκBα: inhibitory protein κBα; IL-1β: interleukin-Iβ; LD_50_: median lethal dose; LDL: low density lipoprotein; MMP-9: matrix metalloproteinase-9; NF-κB: nuclear factor-κB; OA: osteoarthritis; PTP: permeability transition pore; QSAR: quantitative structure-activity relationships; SAR: structure-activity relationship; TNF-α: tumor necrosis factor-α

## Competing interests

The authors declare that they have no competing interests.

## Authors' contributions

KHL and HI conceived and drafted the paper. QS and TA provided technical assistance. SMN edited the manuscript.
